# Capybaras (*Hydrochoerus hydrochaeris*) in the City: Understanding Urban Coexistence, Management Strategies and the Animal Welfare Implications

**DOI:** 10.3390/ani16010113

**Published:** 2025-12-31

**Authors:** Katia M. Nunes Sayn, Maria José Hötzel, Selene S. C. Nogueira

**Affiliations:** 1 Laboratory of Applied Ethology and Animal Welfare, Federal University of Santa Catarina, Florianópolis 88034-001, SC, Brazil; kamedved@gmail.com; 2 Departamento de Ciências Biológicas, Universidade Estadual de Santa Cruz, Ilhéus 45662-900, BA, Brazil; selene@uesc.br

**Keywords:** human–wildlife coexistence, One Welfare, wildlife management

## Abstract

In Brazil, the presence of capybaras (*Hydrochoerus hydrochaeris*) in urban environments has raised concerns due to ecological, sanitary and coexistence challenges. This study investigates the growing presence of capybaras on Santa Catarina Island, Brazil, exploring their integration into urban areas. We interviewed 1505 residents of the Island to understand capybara distribution, public perceptions and associated risks. Beliefs about management strategies were also assessed. The findings reveal that capybaras are present in all neighborhoods of Florianópolis, the sole city in the Island. Participants expressed positive views of capybaras, describing them as “beautiful” and “cute” and showing concerns for their well-being in urban environments. Despite these positive perceptions, participants demonstrated limited knowledge about capybara biology (88%) and potential problems, such as disease transmission (37%), traffic accidents (10%), pet conflicts (56%) and garden damage (42%). Most respondents agreed with the statements: “Capybaras are part of nature and should be left undisturbed, even in urban areas” (70%) and “It is important for people to see capybaras in the city, as long as their population is not causing problems” (82%). After risk awareness, more participants rated these issues as highly relevant: disease transmission (97%), destruction of urban gardens (77%), pet incidents (92%) and traffic accidents (98%). This led to greater acceptance of population control, though elimination remained unpopular. Sociodemographic factors influenced opinions. Addressing knowledge gaps can foster ethical, supported management strategies. This case places Florianópolis within a broader South American phenomenon of urban capybara conflicts, highlighting the global challenge of managing human–wildlife interactions in cities.

## Simple Summary

Capybaras are increasingly common in urban areas of Florianópolis, Brazil, raising concerns about public health, sanitation, and human–wildlife coexistence. We surveyed 1505 residents to map capybara distribution, assess public perceptions, and evaluate whether information about urban risks influences support for management actions. Capybaras were reported in all neighborhoods and were generally viewed positively, often described as attractive or charismatic. However, awareness of associated risks, such as tick-borne diseases and traffic accidents, was limited. After receiving information about these issues, participants rated the risks as more important and showed increased support for population management, although lethal control remained unpopular. These results highlight the role of public knowledge in shaping attitudes toward wildlife management and support the use of integrative frameworks, such as One Welfare, to promote ethical and socially acceptable strategies for urban coexistence.

## 1. Introduction

The population of capybaras (*Hydrochoerus hydrochaeris*), a species endemic to South America, has increased due to its high reproduction rates [1,2,3,4], linked to contributing factors such as the loss of natural predators, such as the jaguar (*Panthera onca*), whose area of occupancy has declined by about 55% across its historical range [5], the abundance of food in agricultural and urban environments, and the species’ tolerance to human presence [6,7]. This population growth is closely associated with intensive anthropogenic activities that have drastically altered the landscapes and habitats where capybaras thrive. The reduction in natural spaces, coupled with the expansion of agricultural and urban areas, has led capybaras to coexist with humans in these environments. They typically occupy areas that include the three main components for their survival: pasture for feeding, water bodies, and shelter [8]. The species’ prolificacy, dietary plasticity, and adaptability to human-altered areas have led to the perception of capybaras as a pest species in some regions [9,10,11]. In these environments, capybara populations can reach large sizes, with herds exceeding 100 individuals [12]. Conflicts with humans may arise, resulting in traffic accidents [13], damage to private property, invasion of public and private spaces, destruction of crops [6,8,9,14,15,16,17,18] and the maintenance and spread of tick-borne diseases [15,19,20,21,22].

Human–wildlife conflict has become increasingly prevalent across all continents [23]. Wildlife managers and animal welfare researchers, while acknowledging the necessity of preserving biodiversity and valuing animal rights and welfare, often disagree on the management of animals classified as pests [24,25]. Invasive species are recognized as one of the primary threats to biodiversity [26,27]. Even native species can become functionally invasive when introduced or overabundant, disrupting ecological balances and threatening native biodiversity, as observed with capybaras (*H. hydrochaeris*) on Anchieta Island, Brazil [28]. For wildlife managers, prioritizing conservation plans and controlling vertebrate pests often outweigh the concerns for the well-being of superabundant species. Consequently, the eradication or control of abundance of these abundant species, such as capybaras, is frequently regarded as a necessary measure to prevent the extinction of other native species [27,28,29]. Conversely, researchers focused on animal welfare tend to prioritize the individual interests of animals over ecosystem conservation, generally advocating for less extreme measures [24].

Public opinion regarding the challenges associated with managing vertebrate pests also plays a crucial role in shaping control policies [30]. Popular support has been shown to influence environmental legislation significantly, to the extent of altering the status of invasive species, such as reclassifying them as native in Australia [31,32]. Given the considerable impact that society has on these issues and its ability to shape environmental policy, it is essential to conduct more in-depth studies on public perceptions regarding these scenarios, especially since human–wildlife coexistence is a dynamic process that involves ongoing negotiations among different stakeholder groups [33].

In recent years, the presence of capybaras in the city of Florianópolis, the sole city occupying Santa Catarina Island in southern Brazil, has increased. However, despite their growing visibility throughout the city, there is no formal record of the capybara population, nor an assessment of potential issues they may be causing, or the opinions of residents on this matter. Therefore, we aimed to provide an initial characterization of the species’ presence in Florianópolis, based on reports from research participants. We also sought to identify potential issues associated with their presence, assess public perceptions regarding capybaras and possible management actions, and examine whether providing information about the species could influence attitudes toward human–capybara coexistence.

## 2. Materials and Methods

### 2.1. Survey and Data Collection

Ethical approval was obtained from the Federal University of Santa Catarina (UFSC), Brazil (CAAE nº 70071023.6.0000.0121; 10 July 2023). Data collection was conducted between July and September 2023 using a self-administered questionnaire carried out through Google Forms Online platform. Participants were recruited via social media by disseminating a survey link. The recruitment channel consisted of sponsored advertisements displayed in Instagram and Facebook Stories, which invited users to complete a survey about wildlife on Santa Catarina Island. The ad read: “Do you live in Florianópolis? We want to hear your opinion about wildlife on the island! Take this survey and be part of a public opinion study with Florianópolis residents”. The target audience parameters were location (city of Florianópolis), age (18 to 65 years—maximum selectable range), gender (all), interests (a wide range to reach a diverse audience—nature, environment, Florianópolis, the sea, hiking, quality of life, entertainment, pets, mountains). The estimated audience size ranged from 296,100 to 348,400 social media accounts. According to the platforms’ analytics, the sponsored advertisements generated over 3700 link clicks. The survey link was also shared via the authors’ WhatsApp contacts residing on the island, with encouragement to further disseminate it.

Only participants aged 18 years or older were included. Geographic delimitation was ensured by restricting the study to neighborhoods of Florianópolis located on Santa Catarina Island, as specified in the questionnaire title and response options. The identity of the participants was not required.

Participation required reading and accepting an informed consent form before starting the questionnaire. The text clarified the study’s purpose, guaranteed anonymity, stated that participants would remain unidentified even to researchers, and that data would be used exclusively for scientific purposes. It also stated that participation involved no risk, offered no compensation, and could be withdrawn at any time without consequences, simply by not submitting the form. The questionnaire included 16 closed questions and two open-ended questions related to the objective of the study and socio demographic questions to characterize the participants.

The questions addressed the presence of capybaras in the city, knowledge about the species and opinions about the need or not to control the capybara population.

Although focused on capybaras, the survey included questions about toucans (*Ramphastos* spp.), marmosets (*Callithrix jacchus*), and caimans (*Caiman latirostris*) to broaden stakeholder engagement and frame the study as addressing urban wildlife more generally. These species were selected because they are well-known and frequently observed on the island: toucans and caimans are native, while marmosets, although introduced, have become widespread and highly adapted to the local environment. This approach aimed to capture a broader range of perspectives, reducing bias from highly engaged or extreme viewpoints and including voices beyond institutional or specialist groups [34].

AI was used exclusively to improve English grammar and clarity.

### 2.2. Structure and Organization of the Questionnaire

The questionnaire was developed as a semi-structured instrument addressing multiple dimensions of public perceptions toward urban capybara management. As it was not intended to measure a single latent construct, internal consistency metrics such as Cronbach’s α were not applied [35]. Content validity was ensured through expert review and pre-testing to assess clarity and relevance. Open-ended responses were translated from Portuguese to English using a translation–back-translation procedure performed by two independent bilingual researchers, with discrepancies resolved by consensus.

The questionnaire had three parts:

Part 1. Information on the presence of capybaras in Florianópolis

Participants were asked whether they had observed capybaras in Florianópolis in 2023. If yes, they were prompted to specify the type of location (e.g., streets, forests, streams, beaches), the neighborhoods, group composition (solitary, adults or with offspring) and the year they first observed capybaras in the city (2023, 2022, 2021, 2020, 2019 or earlier).

Part 2. Level of knowledge and beliefs towards wild animals

Participants were asked to self-assess their level of knowledge about the biology of the wild animals included in the survey. The question specified that “knowledge” referred to understanding of the species’ natural history, including habitat, diet, natural reproduction, and various aspects of their interactions with other animals and with humans in both natural and urban environments. Participants rated their perceived knowledge on a 5-point scale (from no knowledge to extensive knowledge). They were also asked to indicate their level of agreement, using a 5-point Likert scale ranging from 0 (totally disagree) to 4 (totally agree), with an additional option 5 (“I do not know or have no opinion”), regarding statements about the presence of free-ranging populations of these animals in Florianópolis: (1) “It is important for people to see capybaras in the city, as long as their population is not causing problems”; (2) “I like to see this animal in Florianópolis (or if you have never seen it, you would like to see it)”; (3) “This animal is part of nature and should be left alone, even in urban areas”; (4) “This animal causes problems in urban environments, it is necessary to take measures to reduce its population in Florianópolis”; and (5) “This animal causes problems in urban environments, it is necessary to take measures to eliminate its population from Florianópolis”.

Participants answered two open-ended questions: “What problems do you know or think capybaras can cause in urban environments” and “Give a report if you know of any problems caused by capybaras in Florianópolis”.

**Figure 1:**
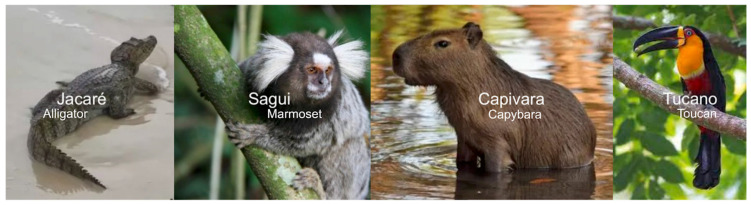
Photos of adult animals of the species alligator, marmoset, capybara, and toucan that precede the chart in which participants had to mark the adjectives that represented how they evaluated each of these wild animals. The picture includes the name of each animal written in Portuguese in front of the corresponding photo, followed by the English name.

The following question required a binary response about the participant’s knowledge before the survey (knew; did not know) on potential problems caused by capybaras in urban environments (disease transmission to humans; damage to urban or domestic gardens; pet-related accidents; traffic accidents; illegal hunting; hindering swimming/navigation). All listed problems were then confirmed as possible, and participants rated their importance on a 3-point Likert scale (0 = not important to 3 = very important). Next, participants viewed photos of adult individuals of each species (Figure 1) and evaluated them based on six attributes: cute, dangerous, beautiful, disgusting, graceful and destructive. The selected attributes were inspired by previous research on affective and cognitive responses to wildlife [36].

Photos of adult animals of the species alligator, marmoset, capybara, and toucan that precede the chart in which participants had to mark the adjectives that represented how they evaluated each of these wild animals. The picture includes the name of each animal written in Portuguese in front of the corresponding photo, followed by the English name.

To conclude, participants were asked again to respond to the same four statements about free-ranging capybaras in Florianópolis: (1) “It is important for people to see capybaras in the city, as long as their population is not causing problems”; (2) “I like to see this animal in Florianópolis (or if you have never seen it, you would like to see it)”; (3) “This animal is part of nature and should be left alone, even in urban areas”; (4) “This animal causes problems in urban environments, it is necessary to take measures to reduce its population in Florianópolis”; and (5) “This animal causes problems in urban environments, it is necessary to take measures to eliminate its population from Florianópolis”.

Part 3. Participant socio-demographic information

The next four questions collected socio-demographic data: self-identified gender (female, male, other, or prefer not to answer), age group (18–25, 26–30, 31–40, 41–50, 51–60, 61–70, or 71 years and older), education level (incomplete school education, completed school education, or completed/ongoing university education), and, if applicable, graduation course.

### 2.3. Statistical Analysis

Of the 1510 completed questionnaires, five were excluded due to incomplete responses, resulting in 1505 valid cases. Based on the population of Florianópolis (537,211 inhabitants [37]), an approximate margin of error of 2.52% was estimated using the standard formula for proportions under maximum variability [38]. Given the self-selected nature of the sample, this estimate should be interpreted with caution, as discussed in Kalton and Flores-Cervantes (2003) [39].

Descriptive statistics for the responses were calculated using Google Sheets, while all other statistical analyses were performed using R Studio software version 4.4.1 [40]. For statistical analysis, participants were classified as having university education (complete or ongoing) or no university education. Age 61–70 and over 71 years old were grouped, due to the low number of participants in these categories. University courses were grouped into fields of science (Engineering and technology; Natural sciences and health science; Social sciences; Non-graduates) due to the high number of graduation courses mentioned. Likert responses to attribute a level of agreement with the statements presented in the survey were grouped into ‘disagree’ (totally or partially) and ‘agree’ (totally or partially), and ‘neutral/I do not know or no opinion’.

Given the discrepancy between the sample’s gender proportions (35% men and 63% women) and those of the municipal population (48% men and 52% women), the data were weighted to improve the accuracy of the estimates. Post-stratification weighting factors of 1.36 for men and 0.82 for women; these were calculated as the ratio between population proportions and sample proportions for each gender and applied following established survey methodology [38]. These weights were incorporated into the McNemar test analyses.

A proportional symbol map (bubble map) was created using data from survey responses that indicated locations where participants had sighted capybaras in Florianópolis. Each symbol represents one or more reports of capybara presence, with symbol size proportional to the number of sightings in each neighborhood. The map was generated in RStudio [39] using the R packages 1.4.1717 sf [41], geobr [42] and ggplot2 [43].

The levels of agreement with the four statements about opinion of free-ranging capybara populations presented in the beginning and in the end of the survey were tested with McNemar test. This test analyzes paired dichotomous data and provides insights into whether there is a significant difference comparing agreement before and after the information provided in the survey. For the first comparison in Table S1, pre- and post-information attitudes were assessed using two conceptually related but non-identical statements, reflecting a shift from a general coexistence perspective to affective appreciation of capybaras. The remaining comparisons involve identical statements assessed before and after information provision. The test was specifically applied to answer the question: Does knowledge of potential urban issues related to capybaras influence people’s opinions on the necessity of population control?

Evaluation of the animals in the chart with adjectives (cute, dangerous, beautiful, disgusting, graceful, destructive) was considered as salient beliefs towards the animal, as described by Manfredo (2008) [44]. With three positive and three negative adjectives available to rate the animals on the chart, a balance of the selections was calculated: each positive adjective selected was assigned a value of +1 and each negative adjective a value of −1. A positive final score indicated a positive belief toward the animal; a score of zero indicated a neutral belief and a negative score indicated a negative belief.

Pearson’s chi-square tests were performed in R to examine associations between participants’ beliefs, gender, age, level of knowledge, and field of science. When appropriate, Multiple Correspondence Analysis (MCA) was used to explore multivariate patterns of association among these variables and participants’ acceptability of statements regarding maintaining, reducing, or eliminating capybara populations in the city.

MCA is based on chi-square distances calculated from an indicator matrix and represents variable categories as points in a reduced-dimensional space, with categories farther from the origin contributing more strongly to the observed variance and closely positioned categories tending to co-occur. The quality of representation of variable categories on the factorial dimensions was assessed using squared cosine values (cos^2^), following standard procedures [45]. Interpretation focused on categories showing both high cos^2^ and high contribution values, as these reflect the most ecologically and socially meaningful attitude patterns. Analyses were conducted using the R packages FactoMineR, factoextra, and gplots.

The open-ended responses from the questionnaire were analyzed using the codebook thematic analysis (TA) approach [46]. This process involved data familiarization, iterative coding guided by a predefined framework, and systematic organization in a Google Sheets table. Themes were identified and refined to encapsulate shared meanings and patterns within the data, serving as domain summaries. Responses spanning multiple themes were coded into all relevant categories and illustrative quotes were used to ensure transparency and contextual relevance. The analyses were conducted by KMS and MJH, who compared their findings and resolved discrepancies through discussion until consensus was achieved. A total of 1009 responses were analyzed to the question, “What problems do you know or think capybaras can cause in urban environments?” and 235 responses to the question, “Please report any problems caused by capybaras in Florianópolis.” Not all participants provided answers, and some responses were excluded due to lack of meaningful content. Representative quotes included in the paper were translated from Portuguese to English by KMS and revised by MJH.

## 3. Results

### 3.1. Socio-Demographic Characterization

Most participants were female, younger than 40 years old and had ongoing or completed university education (Table 1). The educational level of participants was consistent with the adult population of Florianópolis, according to preliminary data from the 2022 Brazilian Census [47], which shows that, among residents aged 25 years and older, 34% have completed upper secondary education and have some higher education, and 42% have completed higher education. In our sample, 79% of participants reported having completed or ongoing higher education, a proportion very close to the 76% observed in the municipality.

### 3.2. Capybaras on the Island

**Figure 2:**
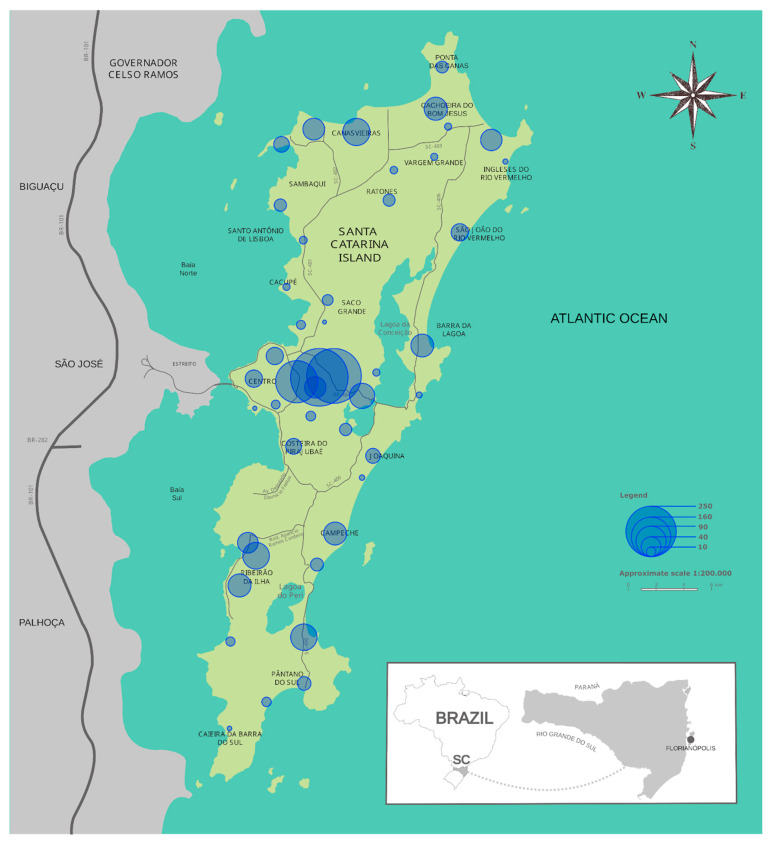
Proportional symbol map showing the neighborhoods with capybara sightings reported by participants in the Island of Santa Catarina, Florianópolis, Santa Catarina State, Brazil.

In 2023, participants reported capybara sightings in all neighborhoods of Florianópolis (Figure 2). The most frequent were groups with offspring (37%), followed by both groups and solitary animals (19%), solitary individuals (16%) and adult groups (15%). Fourteen percent reported no sightings.

When asked about the year, they first noticed capybaras in the city, 15% reported 2019 or earlier, 8% in 2020, 12% in 2021, 16% in 2022, and 10% in 2023; 31% did not know and 9% had not seen capybaras. Although 15% reported sightings in 2019 or earlier, most observations (45%) occurred between 2020 and 2023.

Regarding sighting locations, 34% reported streets or sidewalks, 50% mangroves, lagoons, or wetlands, 25% wooded areas, 48% rivers or streams, 10% beaches, 4% sewage areas, 3% personal or friends’ gardens, 1% parks, and 13% did not recall. As multiple responses were allowed, the total exceeded 100%.

When self-assessing their knowledge of capybara biology, 36% reported superficial knowledge, 52% some knowledge, and 12% a lot of knowledge.

### 3.3. Issues Regarding Capybaras in Urban Environments

The first open-ended question, answered by 67% of participants, revealed 10 primary themes: zoonoses spread, traffic accidents, capybara welfare concerns, incidents with pets and humans, overpopulation, ecosystem disturbance, garden or crop damage, droppings and illegal hunting. The second question, answered by 16%, identified five themes: traffic accidents (9%), tick or tick-borne disease spread (2%), accidents involving people or pets (2%), garden or crop damage (2%), and population growth (1%).

#### 3.3.1. Public Perceptions of Potential Problems

Zoonoses were the main concern (32%), especially Brazilian Spotted Fever (BSF) and other tick-borne diseases. Traffic accidents followed (29%), citing risks of collisions and run-overs (e.g., Participant 195: “They can invade some streets and roads causing accidents and they can be carriers of several species of ticks that can transmit diseases to humans”). Concerns about capybara well-being were raised by 10% of participants, citing urban risks. Participant 550 stated, “The greatest risk is to the capybara populations themselves, as they face challenges in urban environments.” Participant 599 noted, “The urban environment causes problems for the movement of capybaras, especially near streams and mangroves”.

Incidents with pets and humans were mentioned by 7%, while 6% reported no problems were caused by capybaras. However, overpopulation was a concern for 5% of participants, with respondents noting the uncontrolled growth of capybara population. Concerns about disturbance of ecosystems were mentioned by 4%, destruction of gardens or crops was noted by 3%, and dropping issues were mentioned by 1% of the participants. Finally, illegal hunting in the urban environment was raised as a concern to six participants (<1%).

#### 3.3.2. Reported Problems in Florianópolis

Six participants mentioned ecosystem disturbance and five raised public policy issues, critiquing inadequate planning or resources for managing capybara problems. Participant 599 stated, “The problems are not caused by the capybara but by lack of urban planning. For example, traffic accidents can occur”. Three participants noted issues with droppings, one reported navigation problems, and another mentioned illegal hunting.

#### 3.3.3. Balancing Admiration with Concerns: Public Perceptions and Knowledge Gaps

Several participants expressed nuanced views, balancing admiration for capybaras with management concerns. Participants 661 and 653, for example, acknowledged their native status (“Although they cause urban problems, they are native animals and should be respected as such”; “The ideal would be to protect and allow the animals to thrive while preventing harm to both them and us”).

Despite concerns, participants showed limited understanding of capybara biology and varied awareness of associated risks; 63% recognized zoonotic disease transmission and 83% identified traffic accidents as significant (Table 2).

Participants also exhibited predominantly positive beliefs about capybaras, describing them as ‘beautiful’ (61%), ‘cute’ (54%), and ‘graceful’ (42%). Fewer perceived them as ‘dangerous’ (23%), ‘destructive’ (20%), or ‘disgusting’ (6%). Strong support for peaceful coexistence was evident, as reflected in agreement with the statements ‘Capybaras are part of nature and should be allowed to thrive undisturbed, even in urban environments’ (70%) and ‘It is important for people to see capybaras in the city, as long as their population does not cause problems’ (82%).

McNemar tests revealed significant changes in agreement for all statements evaluated (Table S1), indicating that exposure to information significantly influenced participants’ opinions. Agreement with statements supporting population reduction increased significantly after information was provided (Statements 1–4; χ^2^ ranging from 146.00 to 456.11, all *p* < 0.001), supporting the hypothesis that risk awareness alters management attitudes. Although resistance to population elimination remained high, a significant shift was also detected for this statement (χ^2^ = 174.90, *p* < 0.001), indicating increased consideration of management intervention even for more extreme measures.

**Figure 3:**
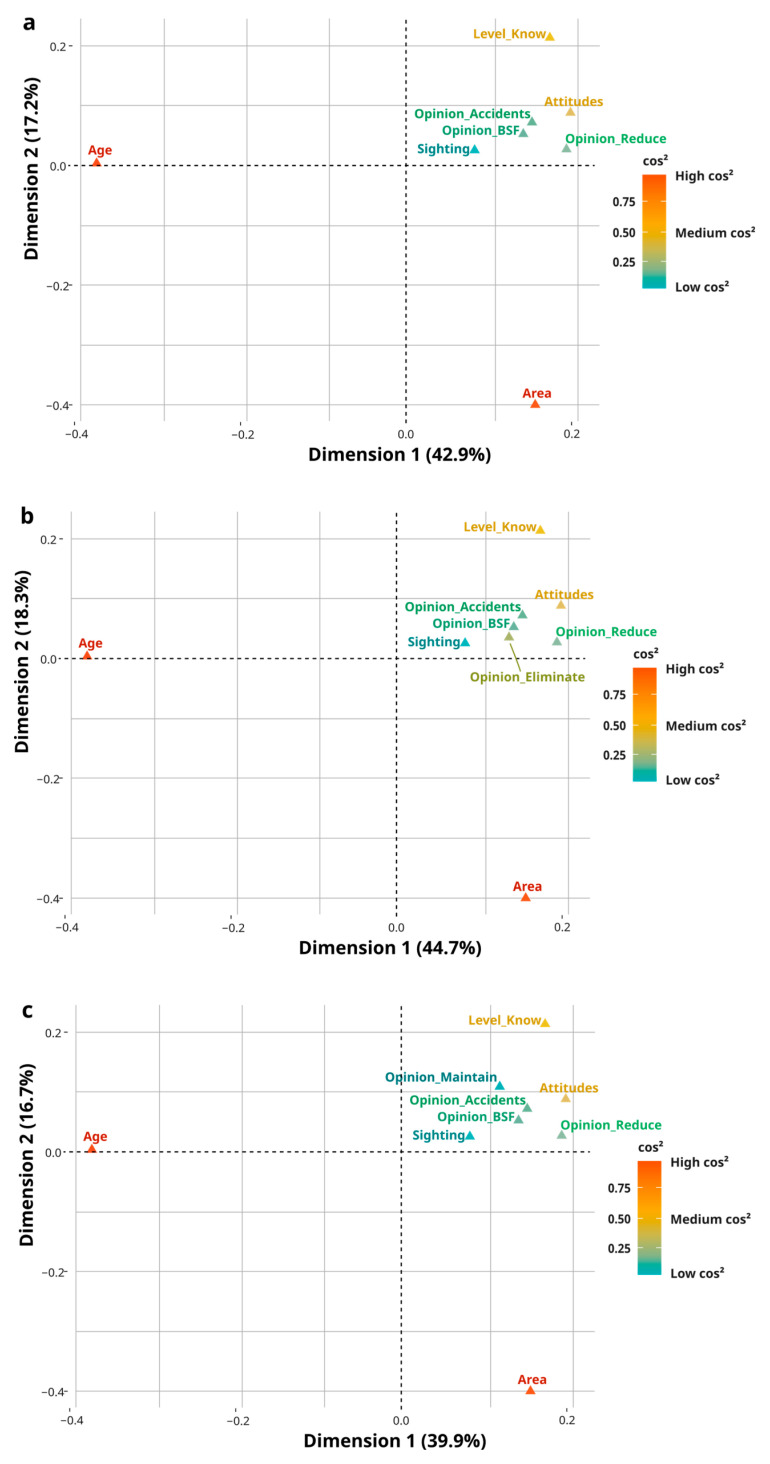
Multiple Correspondence Analysis (MCA) shows the relationships between socio-demographic variables, beliefs, and participants’ attitudes toward capybara population management in Florianópolis. Panels represent opinions regarding (**a**) allowing capybaras to thrive undisturbed, (**b**) reducing the population, and (**c**) eliminating the population. Dimension 1 and Dimension 2 explain the main patterns of variation in responses. Colors indicate the quality of representation of variable categories on the dimensions (cos^2^), with red indicating stronger representation, orange moderate representation, and blue weaker representation.

Associations between beliefs and sociodemographic variables were identified via chi-square tests, when chi-square assumptions were not fully met, Fisher’s exact test was used as a confirmatory analysis. Multiple Correspondence Analysis (MCA) revealed patterns of agreement on population control statements (Table 3). Figure 3 displays relationships between variable categories and the first two dimensions, explaining ~60% of response variation. Dimension 1 relates to beliefs, knowledge, and scientific fields, reflecting broader sociocultural factors, while Dimension 2 is more associated with individual factors such as age and capybara sightings.

Significant associations are observed between socio-demographic variables and agreement or disagreement with management measures (Table S2). Key findings include the significant role of age and beliefs in shaping opinions on capybara management. Older participants (aged 51–70) were less likely to support leaving capybaras undisturbed in urban environments and more likely to agree with the need for population reduction or elimination, though all age groups generally disagreed with extreme measures like eradication.

Participants with Natural Sciences backgrounds expressed more disagreement with leaving capybaras undisturbed compared to non-graduates or those in the fields of Engineering and Technology or Social Sciences. Similarly, they were more inclined to support population reduction.

Knowledge level also shaped opinions: participants with superficial knowledge were less supportive of leaving capybaras undisturbed and more often chose “I don’t know or no opinion” on management questions.

Salient beliefs about capybaras strongly influenced support for management. Positive beliefs aligned with support for leaving capybaras undisturbed and opposition to population control, while negative beliefs correlated with greater support for management, though elimination remained broadly opposed.

Perceived risks also influenced support for management. Participants who rated BSF or traffic accidents as highly important were more likely to support population control, including reduction or elimination, though agreement with elimination remained very low across all groups.

Overall Dimension 1, associated with social and cultural factors, aligns with variable categories like knowledge level and scientific field, shaped by education and academic background. Dimension 2 reflects individual characteristics, such as age and capybara sightings. This distinction captures different layers of public opinion while acknowledging the interplay between individual and contextual factors.

## 4. Discussion

### 4.1. Capybara Distribution and Abundance in the Island of Santa Catarina

Capybaras are distributed across all neighborhoods of Florianópolis, spanning the entire island. This places Florianópolis among other South American cities experiencing similar phenomena, indicating that the presence of capybaras in urban settings constitutes a widespread, continent-scale dynamic [48]. Ecological [12] and legal [49] factors contribute to capybaras’ urban expansion, which can lead to rapid population growth, sometimes quadrupling within a year [12]. The increasing trend in capybara sightings reported by participants, particularly from 2020 to 2023, may reflect population growth and the expansion of occupied habitat [50]. Groups with offspring were frequently observed. Our findings are particularly notable when compared to previous studies [51,52], which documented that capybaras were considered extinct on the island during the 1990s, following the last reported sighting in the 1970s, likely due to hunting pressure. The presence of capybaras on the island is well established [53], and scientific documentation suggests that the current population may have originated either from natural recolonization—given their ability to swim across marine channels [18]—or from captive individuals that escaped from a breeding facility in 2001 [52]. Although increased sightings alone do not confirm population growth, as this is influenced by more complex ecological and demographic factors [54], citizen science initiatives like this one are rapidly gaining popularity as low-cost alternatives for gathering data on species abundance and distribution across large spatial and temporal scales [55].

The distribution of capybara sightings reported by participants aligns with the species’ ecological preferences, with significant observations in mangroves, lagoons, wetlands, and rivers or streams. In anthropogenic wetlands, capybara populations often reach higher population densities than in pristine habitats, due to the favorable conditions for population growth offered by these human-modified environments [56].

The increasing trend in capybara sightings may correlate with growing public awareness of urban wildlife, as similar patterns were observed during the COVID-19 pandemic, when reduced human activity enhanced awareness of urban wildlife [57]. Also, their presence in urban areas can be explained by their habituation to human activity. This adaptation diminishes their natural flight response, leading them to perceive humans as non-threatening and allowing them to be more visible and active in urban environments. While this can facilitate coexistence, it also creates potential challenges, such as conflicts when capybaras venture into heavily human-dominated areas [58]. Adaptations to human-modified landscapes further shape the behavior and risks associated with urban-adapted species [59]. For instance, capybaras often adjust their activity patterns in developed areas, exhibiting reduced home ranges, shorter movements, and behavioral shifts, such as increased nocturnal activity [60]. These changes, while enabling survival in urban settings, can unintentionally heighten risks such as traffic collisions [60].

### 4.2. Human-Capybara Conflict

Scientific literature highlights human–capybara conflicts, including zoonotic disease transmission, crop damage, garden damage, tick contamination, traffic accidents [61] and occasional aggression toward humans and pets [62]. Our findings reflect similar concerns among participants, particularly regarding traffic risks, tick-borne diseases, human and pet encounters, while also revealing empathy for the animals’ well-being in urban settings.

Brazilian Spotted Fever (BSF), the most concerning disease linked to capybaras, plays a central role in these conflicts. It is controversial whether capybaras are amplifiers of the disease. BSF is caused by the bacterium *Rickettsia rickettsii* and transmitted by ticks of the genus *Amblyomma sculptum* [15,21], a three-host parasite that affects capybaras [22]. The spatial and temporal dynamics of urban areas inhabited by capybaras enhance the reproductive capacity of ticks, further exacerbating the disease’s transmission [63]. Between 2000 and 2018, 2090 confirmed cases in humans of BSF were reported in Brazil [64]. In response to these concerns, environmental authorities have authorized or required capybara control measures, prompting studies on population management, including sterilization [65], group removal [66], grass avoidance [67] and environmental adjustments to reduce habitat suitability [12]. These interventions not only address population growth but also aim to mitigate the spread of zoonotic diseases [14]. To our knowledge, there have been no reported cases of BSF in Florianópolis to date.

Traffic accidents involving capybaras, especially during peak nocturnal hours between 18:00 and 21:00 [60] are significant in Brazil, where capybaras rank among the most common animal victims of traffic accidents [68]. This concern is reflected in our findings, where participants frequently mentioned sightings of capybaras on streets or sidewalks and raised traffic accidents as a significant issue. Transportation infrastructure also acts as physical barriers, exacerbating the likelihood of collisions and creating additional hazards for both animals and humans [60]. These observations align closely with broader behavioral and ecological patterns, underscoring the complexities of coexistence in human-dominated spaces.

### 4.3. Public Perceptions and Attitudes

Providing information about urban challenges associated with capybaras, such as ecological and health risks, significantly influenced public opinion. While participants initially held generally positive or neutral views, exposure to information about these risks increased support for population management measures and, to a lesser extent, more extreme control strategies. Similarly, research in Australia [68] demonstrated that public values and beliefs shape the acceptability of wildlife management strategies. Many participants who initially favored leaving capybaras undisturbed became more open to control measures after learning about potential risks. Targeted communication can enhance public support for management [68], reinforcing the view that conservation must account for social-ecological contexts and prioritize participatory approaches [69].

While capybaras were described as “beautiful” and “cute,” these sentiments were tempered by awareness of the challenges they pose in urban environments. In a study conducted in Curitiba’s parks, southern Brazil, visitors appreciated the presence of this species but recognized risks both to the animals (e.g., pollution, hunting) and to humans (e.g., disease transmission) [70]. Our findings also corroborate a study done in Curitiba, where capybaras were simultaneously highly valued and viewed as potential threats to human health [71].

Despite general public appreciation for capybaras, our study identified knowledge gaps about the risks their urban presence poses. Participants’ level of concern did not always align with their awareness of zoonotic disease transmission and the risk of traffic accidents involving capybaras. This discrepancy became more evident when increased awareness of these urban challenges led to a more critical stance on capybaras’ unchecked presence in urban spaces. Public perceptions tend to shift with negative events amplifying public concern [72] revealing the ambivalence between the desire to coexist with urban wildlife and the practical need to address these challenges. Similarly, the marked increase in support for the statement 4 (Table 3) after learning about the problems they might cause emphasizes the influence of information on public perceptions. However, despite increased openness to population control, strong resistance to elimination persisted. Similarly, in Australia, support for koala management rose after information on overpopulation, yet lethal measures remained unpopular [73].

While the public recognizes some risks, limited understanding of the ecological and urban challenges posed by capybaras may hinder support for management efforts. Similar challenges have been observed with other charismatic wildlife, such as koalas, underscoring the importance of public education in bridging the gap between admiration for a species and the recognition of its management needs [73]. Participants with limited knowledge about capybaras tended to express greater uncertainty, especially regarding management.

We can further understand these patterns in public perceptions through the analysis of socio-demographic factors associated with the level of agreement toward capybara population management in urban environments. A background in Natural Sciences was associated with more support for population reduction, which may reflect greater exposure to knowledge of wildlife management and its connection with conservation efforts. This finding resonates with previous research highlighting how academic background and professional training shape individuals’ perspectives on conservation and animal welfare [73]. Also, participants aged 51–70 years were more likely to agree with population reduction measures. A study in the UK also found that age influences attitudes and opinions towards species considered pests, which is often tied to their personal experiences with wildlife conflicts, perceptions of risk, and expectations of public authorities to address nuisances [74].

Positive beliefs toward capybaras fostered support for maintaining the capybara population undisturbed, and negative beliefs were associated with support for reducing or eliminating the population. These findings underscore the significant role that individual perceptions and emotions play in shaping opinions about wildlife management interventions. Beyond factual knowledge, emotional and experiential connections to nature play a key role in shaping pro-environmental behavior [75].

The literature offers important context for how beliefs about capybaras may evolve. Species-specific traits are crucial [76]. Capybaras are viewed as charismatic, which contributes to the public’s resistance to invasive management measures such as population reduction or elimination. Perceptions may shift if capybaras become associated with risks, such as traffic accidents or property damage [74]. Perceptions of risk, even when based on infrequent or minor negative interactions, can strongly influence attitudes [76]. Although Brazilian Spotted Fever (BSF) has not been reported on the island to date, any future cases could intensify concerns and increase support for population control. Indeed, perceived risks from BSF and traffic accidents significantly influenced opinions in this study, with participants who rated these risks as important being more likely to favor population reduction or elimination.

### 4.4. Management Implications

Managing urban capybara populations involves complexities beyond the species itself, as they are embedded in environmental and social systems. This aligns with the One Health framework, which emphasizes the interdependence of human, animal and environmental health. Studies identifying capybaras as reservoirs of zoonotic parasites [12,77] highlight the need to adopt this approach to mitigate risks and promote collective well-being.

Furthermore, addressing the broader challenges posed by capybaras in urban environments can be strengthened by incorporating the One Welfare framework [78], which explicitly emphasizes animal welfare—an aspect highlighted by participants in this study. Integrating these frameworks into wildlife management may foster approaches that are both scientifically grounded and aligned with public values, as they provide a structured way to navigate trade-offs between human health, animal welfare, and environmental sustainability in urban wildlife management [79].

For example, interventions such as habitat modification [12,67], population monitoring [70], fertility control [65], and public education [44,58,70] may align more closely with One Welfare principles than indiscriminate population reduction [26], as they address human safety concerns while preserving animal welfare and ecosystem functions. Even where population control is deemed necessary, the One Welfare framework emphasizes that management actions should be proportionate, welfare-oriented, and transparently justified, recognizing capybaras as sentient animals embedded in complex social and ecological systems. By explicitly balancing these dimensions, One Welfare offers a practical ethical guide for decision-making, rather than a purely conceptual extension of One Health, supporting management approaches that are both socially legitimate and ecologically sustainable.

Urban wildlife presents both challenges and opportunities. While conflicts may occur, the presence of wild animals in cities can strengthen human–nature connections and support mental well-being [80]. As urbanization advances and people grow more disconnected from nature, integrating wildlife offers a chance for reconnection [81]. Realizing these benefits requires coordinated efforts to ensure safe, sustainable coexistence. Urban landscapes should be designed to accommodate wildlife while mitigating conflicts, reducing risks and promoting the well-being of all involved [82]. The acceptability of management strategies for unwanted encounters remains contested [83], and their success depends largely on public perceptions and attitudes. Although this study focuses on capybaras, the approach applies to other superabundant, charismatic urban species that evoke public empathy and influence conservation decisions. By integrating ecological, social, and welfare dimensions, the One Welfare framework provides adaptable, ethically sound, and socially acceptable guidance for urban population management. As conservation embraces a systems-based approach, wildlife management must be understood within its broader social and ecological contexts [70]. Consulting the public to assess appropriate control methods—considering cultural and social factors—helps ensure democratic policies that respect animal rights and public concerns [83].

Based on the identified knowledge gaps and the significant change in attitudes after risk information was provided, the results support management actions such as targeted public education on zoonotic diseases and traffic risks, as well as non-lethal interventions in areas with frequent capybara sightings. The feasibility and economic costs of management options such as sterilization, translocation, or habitat modification can vary widely across urban contexts and were not assessed in this study. These factors should be evaluated in future interdisciplinary research combining ecological monitoring, economic assessment, and institutional analysis to inform operational decision-making.

## 5. Conclusions

Capybaras were reported by respondents across all neighborhoods of Florianópolis, Santa Catarina Island, indicating their widespread presence in the urban environment. Survey results showed that residents generally held positive perceptions of capybaras. However, knowledge about risks associated with urban coexistence, particularly tick-borne diseases, traffic collisions, and interactions with domestic animals, was limited. After receiving information about these risks, participants’ perception of the importance of these issues significantly increased, accompanied by greater support for population management measures, whereas rejection of eradication remained consistent. These findings indicate that public support for management actions is strongly influenced by risk awareness and that non-lethal, socially acceptable approaches are more likely to receive public approval.

## Figures and Tables

**Table 1:** Socio-demographic information of survey participants (n = 1505), 2023.

*Variable*	*n*	*(%)*
*Gender*		
Female	953	63
Male	522	35
Other	14	1
I’d rather not respond	16	1
*Age*		
18 to 25 years old	332	22
26 to 30 years old	243	16
41 to 50 years old	246	17
51 to 60 years old	156	10
61 years old and over	95	6
*Education*		
No university education	323	21
University education complete or ongoing	1182	79

**Table 2:** Participants’ (n: 1505) awareness and opinions about the potential problems caused by capybaras.

	Did Not Know		Very Important	Somewhat Important	Not at All Important
	n	%	%	%	%
Transmission of Brazilian Spotted Fever (BSF)	598	40	86	12	2
Risk of causing traffic accidents	152	10	83	15	2
Zoonotic disease transmission	658	37	83	14	3
They can be run over	68	5	77	19	4
Motivate illegal hunting	757	50	67	23	10
Be bitten by domestic animals	515	34	59	33	8
Bite domestic animals	834	55	53	36	11
Impair navigation and swimming	1113	74	37	40	23
Destruction of crops and herb gardens	636	42	28	48	23
Destruction of gardens	636	42	19	47	34

**Table 3:** Pearson’s Chi-square test results examining the relationship between socio-demographic variables and responses indicating agreement or disagreement with statements on eliminating, reducing, or maintaining the capybara population in Florianópolis, Brazil.

	This Animal is Part of Nature and Should be Left Alone, Even in Urban Areas	This Animal Causes Problems in Urban Environments, It is Necessary to Take Measures to Reduce Its Population in Florianópolis	This Animal Causes Problems in Urban Environments, It is Necessary to Take Measures to Eliminate Its Population from Florianópolis
Variable	*p*-Value	*p*-Value	*p*-Value
Identified Gender	0.6601	0.23	0.07039
Age	0.0002854 **	0.4203	0.002703 **
Field of science	0.02786 **	3.069 × 10^−5^ **	0.5497
Level of knowledge	0.001683 **	0.08264	1.479 × 10^−5^ **
Sighting	0.3831	0.1272	0.03801 **
Beliefs	<2.2 × 10^−16^ **	<2.2 × 10^−16^ **	<2.2 × 10^−16^ **
Opinion (BSF)	4.956 × 10^−14^ **	4.426 × 10^−16^ **	0.0002498 ** Fisher test 4.736 × 10^−5^ **
Opinion (Accidents)	4.143 × 10^−14^ **	<2.2 × 10^−16^ **	3.795 × 10^−6^ **

Numbers followed by ** are statistically significant at the 5% level (*p* value < 0.05).

## Data Availability

The data supporting the findings of this study are available from the corresponding author upon reasonable request.

## Supplementary Materials

The following supporting information can be downloaded at: https://www.mdpi.com/article/10.3390/ani16010113/s1, Table S1: Paired responses to attitude statements before and after access to information; Table S2: Percentages of agreement, disagreement, or uncertainty (“I do not know or no opinion”) across socio-demographic groups and other variables regarding statements about eliminating, reducing, or maintaining the capybara population in Florianópolis, Brazil. Results are based on significant associations identified through Multiple Correspondence Analysis (MCA).
