# Supplementary material for: Capybaras (Hydrochoerus hydrochaeris) in the City: Understanding Urban Coexistence, Management Strategies and the Animal Welfare Implications
Source: Animals (Basel). 2025 Dec 31;16(1):113. doi: 10.3390/ani16010113 (PMC12784735; doi:10.3390/ani16010113)
Supplement: Supplementary file 1 [file animals-16-00113-s001.zip › animals-4062132-supplementary.pdf]

SUPPLEMENTARY MATERIAL

Table S1. Paired responses to attitude statements before and after access to information

Statement 1 (non-identical pre- and post-information statements)
Before: *"It is important for people to see capybaras in the city, as long as their population is not causing problems."*
After: *"I like to see this animal in Florianópolis (or, if you have never seen it, you would like to see it)."*

**Before \ After**	**Agree**	**Disagree**
**Agree**	918	144
**Disagree**	144	126

Statement 2 (identical before and after)
"Capybaras are part of nature and should be allowed to thrive undisturbed, even in urban environments."

**Before \ After**	**Agree**	**Disagree**
**Agree**	758	233
**Disagree**	39	213

Statement 3 (identical before and after)
"Capybaras cause problems in urban environments, and measures must be taken to reduce their population in Florianópolis."

**Before \ After**	**Agree**	**Disagree**
**Agree**	362	66
**Disagree**	161	613

Statement 4 (identical before and after)
"Capybaras cause problems in urban environments, and measures must be taken to eliminate their population in Florianópolis."

**Before \ After**	**Agree**	**Disagree**
**Agree**	86	49

**Disagree**	40	1174

McNemar test results are based on gender-weighted data. Absolute frequencies represent unweighted counts. Statement 1 compares conceptually related but non-identical items, whereas Statements 2–4 involve identical wording assessed before and after information provision.

Table S2. Percentages of agreement, disagreement, or uncertainty ("I do not know or no opinion") across socio-demographic groups and other variables regarding statements about eliminating, reducing, or maintaining the capybara population in Florianópolis, Brazil. Results are based on significant associations identified through Multiple Correspondence Analysis (MCA)

		This animal is part of nature and should be left alone, even in urban areas			This animal causes problems in urban environments, it is necessary to take measures to reduce its population in Florianópolis			This animal causes problems in urban environments, it is necessary to take measures to eliminate its population from Florianópolis		
Variable		Agree	Disagree	No opinion	Agree	Disagree	No opinion	Agree	Disagree	No opinion
	Overall percentage	54	30	16	36	46	18	8	83	9
		(%)	(%)	(%)	(%)	(%)	(%)	(%)	(%)	(%)
Age	18 to 25 years old	56.0	26.5	17.5	33.4	47.9	18.7	3.9	88.9	7.2
	26 to 30 years old	60.1	25.1	14.8	34.6	42.8	22.6	7.4	84.8	7.8
	31 to 40 years old	53.8	27.5	18.7	35.1	47.3	17.6	7.2	82.7	10.2
	41 to 50 years old	55.3	30.5	14.2	37.4	45.1	17.5	11.8	78.5	9.8
	51 to 60 years old	45.5	43.6	10.9	41.7	45.5	12.8	14.7	78.2	7.1
	61 to 70 years old	43.8	46.3	10.0	45.0	42.5	12.5	13.8	77.5	8.8
	71 years old and over	73.3	20.0	6.7	40.0	40.0	20.0	0.0	93.3	6.7
Fields of science	Natural sciences	47.3	37.1	15.6	47.8	37.4	14.8	10.2	81.7	8.1
	Engineering and	56.7	25.7	17.5	33.3	50.9	15.8	7.0	82.5	10.5

	technology									
	Social sciences	55.7	28.3	16.0	33.2	47.6	19.2	7.2	84.8	8.0
	Non-graduates	58.5	27.2	14.2	30.7	49.5	19.8	9.0	81.5	9.6
Level of knowledge	Superficial knowledge	48.5	31.5	19.9	35.2	45.2	19.6	10.5	76.8	12.7
	Some knowledge	57.1	29.0	14.0	36.2	45.4	18.4	6.5	86.9	6.6
	A lot of knowledge	60.2	29.5	10.2	39.8	50.0	10.2	9.7	85.2	5.1
Sighting	I saw	54.3	30.6	15.1	37.5	45.5	17.1	7.9	84.3	7.8
	I did not see	54.7	27.6	17.7	32.1	47.1	20.7	9.6	78.7	11.7
Beliefs	Positive	63.7	21.2	15.1	27.2	54.4	18.4	3.5	90.6	5.9
	Neutral	37.8	38.5	23.7	51.9	22.4	25.6	9.6	69.9	20.5
	Negative	20.3	66.7	13.0	69.7	20.3	10.0	30.7	55.4	13.9
Opinion (BSF)	Very important	50.1	33.2	16.7	40.1	41.4	18.4	9.5	81.3	9.2
	Somewhat important	78.2	11.7	10.1	12.8	71.5	15.6	1.7	92.7	5.6
	Not at all important	88.6	5.7	5.7	14.3	77.1	8.6	0.0	97.1	2.9
Opinion (Accidents)	Very important	49.0	34.2	16.8	40.8	39.9	19.2	9.6	81.6	8.8
	Somewhat important	68.7	18.0	13.4	24.6	60.6	14.8	2.8	89.4	7.7
	Not at all important	88.7	6.5	4.8	4.8	88.7	6.5welfare	0.0	100.0	0.0

Sections with a gray background are statistically significant at the 5% level (p-value < 0.05) according to the Multiple Correspondence Analysis (MCA) test.
